# Risk adapted approach: How to treat splenic marginal zone lymphoma in resource-poor settings? - The real-life experience of a Brazilian cancer treatment center

**DOI:** 10.1186/s12885-020-07204-6

**Published:** 2020-08-03

**Authors:** Luís Alberto de Pádua Covas Lage, Felipe Faganelli Caboclo dos Santos, Débora Levy, Frederico Rafael Moreira, Samuel Campanelli Freitas Couto, Hebert Fabrício Culler, Renata de Oliveira Costa, Vanderson Rocha, Juliana Pereira

**Affiliations:** 1 Department of Hematology, Hemotherapy and Cell Therapy of Medicine School, Laboratory of Medical Investigation in Pathogenesis and Directed Therapy in Onco-Immuno-Hematology (LIM-31), Sao Paulo University (FMUSP), Rua Maranhão, número 300, apartamento 13 - São Caetano do Sul, São Paulo (SP), 09541-000 Brazil; 2 Department of Hematology, Hemotherapy and Cell Therapy of Medicine School, Sao Paulo University (FMUSP), São Paulo, Brazil; 3 Department of Hematology, Hemotherapy and Cell Therapy of Medicine School, Laboratory of Medical Investigation 19 (LIM-19), Sao Paulo University (FMUSP), São Paulo, Brazil; 4 Statistical, Department of Hematology, Hemotherapy and Cell Therapy of Medicine School, Sao Paulo University (FMUSP), São Paulo, Brazil; 5 Department of Hematology and Hemotherapy, Centro Universitário Lusíadas, Santos, Sao Paulo, Brazil; 6 Pró-Sangue Foundation (Sao Paulo Blood Bank), São Paulo, Brazil; 7 Churchill Hospital, Oxford University, Oxford, UK

**Keywords:** Splenic marginal zone lymphoma, Prognostic factors, Splenectomy, Rituximab, Poor-resource settings

## Abstract

**Background:**

Splenic marginal zone lymphoma (SMZL) is a rare lymphoid B-cell malignant neoplasm with primary involvement of the spleen. It is a chronic disease, of indolent behavior and prolonged survival. However, 25% of cases have higher biological aggressiveness, propensity for histological transformation to high grade B-cell non-Hodgkin lymphoma and shortened survival. Recognition of these cases of reserved outcome is important for selecting a risk-adapted therapeutic approach in a resource-poor settings.

**Methods:**

We described clinical and epidemiological characteristics, survival analysis and prognostic factors in a retrospective cohort of 39 SMZL patients, treated in Latin America.

**Results:**

We observed a predominance of female (71.8%), median age of 63 years and higher incidence of B symptoms (56.4%) and extra-splenic involvement (87.1%) than in European and North-American series. With a median follow-up of 8.7 years (0.6-20.2 years), estimated 5-year overall survival (OS) and progression-free survival (PFS) were 76.9% and 63.7%, respectively. Factors with adverse prognostic impact on OS and PFS were Hb < 100 g/L, platelet count < 100 x 10^9^/L, albumin < 3.5 g/dL, LDH > 480 U/L and high-risk Arcaini and SMZL/WG scores. Despite a relative low number of patients, no superiority was observed among the therapeutic regimens used including rituximab monotherapy, splenectomy and cytotoxic chemotherapy.

**Conclusion:**

Therefore, in resource-poor settings, where access to immunotherapy is not universal for all SMZL patients, we suggest that first-line should consist on rituximab therapy for elderly patients or with high surgical risk or with at least 1 risk factor identified in our study. Remainders can be safely managed with splenectomy.

## Background

Splenic marginal zone lymphoma (SMZL) is a malignant neoplasm with primary involvement of the spleen, characterized by the proliferation of small B-cell lymphocytes, predominantly in the spleen white pulp, with nodular histological arrangement and indolent behavior [1, 2]. It represents 2% of all non-Hodgkin's lymphomas (NHL), but is the most common histological subtype of splenic primary lymphoma [2]. Usually, patients have massive splenomegaly, peripheral blood cytopenias, and absence of peripheral lymphadenopathy, except for splenic hilum [3]. There is no extranodal involvement, except for the liver and bone marrow. About 50%-60% of patients have bone marrow infiltration and up to 25% may have circulating phase, represented by mild or moderate lymphocytosis. Presence of B symptoms and high levels of lactic dehydrogenase (LDH) are uncommon [4]. Presence of paraproteinemia, usually IgM kappa and autoimmune phenomena occur in up to one third of patients [5].

SMZL arises from marginal zone memory B-cells and probably has a post-germinal origin [6]. Chronic antigenic stimulation of B-cell receptor initially induces polyclonal and later monoclonal B lymphocyte expansion, leading to malignant transformation [6]. In up to 36% of cases, this stimulus is represented by chronic hepatitis C virus infection [7]. Molecularly, SMZL is characterized by dysregulation of nuclear factor kappa-B and mutations affecting the *NOTCH*, *KLF2* (Kruppel like factor 2) and *PTPRD* (receptor-type protein tyrosine phosphatase delta) pathways [8].

Considered as an indolent and incurable disease, its treatment is only recommended in symptomatic cases, represented by massive visceromegaly, severe cytopenias, presence of constitutional symptoms or autoimmune phenomena unresponsive to steroids [9]. Therapeutic options for SMZL include splenectomy, monotherapy with anti-CD20 monoclonal antibody or combination therapy, including rituximab and classic chemotherapeutic agents [10, 11]. In the last ten years, several studies have shown superiority of rituximab therapy, either alone or in combination [12–14], but, in resource-poor countries rituximab is not a drug of universal access, making splenectomy still widely used in these particular settings.

The majority of SMZL patients have a disease with favorable prognosis, with a median overall survival (OS) exceeding 10 years, despite the use of specific treatment [15, 16]. However, SMZL prognosis is heterogeneous, and about 20%-30% of the cases show a more aggressive clinical course with a median OS of only 4 years. Histological transformation to high grade B-cell lymphoma may occur in 10%-15% of cases as part of the natural history of this tumor [15, 17]. Thus, the identification of patients with unfavorable outcome becomes necessary for better risk stratification and selection of appropriate therapy.

Clinical and laboratory factors with prognostic impact to guide therapy have been described by different American and European groups using different prognostic scores [15, 18]. Arcaini et al. created a score capable of predicting SMZL prognosis, using a combination of hemoglobin < 120 g/L, elevated LDH and albumin < 3.5 g/dL [18]. A study conducted by the *Splenic Marginal Zone Lymphoma Working Group (SMZL-WG)*, involving 593 patients from different centers, identified Hb < 95 g/L, platelets < 80 x 10^9^/L, elevated LDH and the presence of extra-hilar lymphadenopathy as variables associated with adverse prognosis, representing another widely used prognostic score in clinical practice [15].

In Latin America, particularly in Brazil, there is scarce information regarding SMZL epidemiological data, as well as evolution and prognostic factors that influence survival of these population. The Brazilian national public health system offers limited options to treat these patients. For the most part, anti-CD20 antibodies such as rituximab are not available in public services for SMZL patients. In this context, often the first therapeutic option is splenectomy. Although still a good option for patients with low surgical risk, in elderly patients or in patients with severe comorbidities, splenectomy may be associated with a high risk of morbidity and mortality. In SMZL patients with more aggressive disease, the new treatment progression-free interval can be quite short when using splenectomy. In these scenarios, rituximab therapy may be applied with greater clinical benefit.

In this study, we aimed to report the clinical-laboratory and epidemiological findings of 39 Brazilian patients with SMZL followed at a single referral center for cancer treatment, as well as to determine factors related to adverse prognosis and to propose a rational risk-adjusted therapeutic strategy, considering scenarios where access to monoclonal antibody therapy is limited.

## Methods

### Design of study

This is a unicentric, retrospective and observational study conducted at Instituto do Câncer do Estado de São Paulo (ICESP) and Hospital das Clínicas da Faculdade de Medicina de São Paulo (HC-FMUSP), Brazil. The study was approved by the local Ethics Committee in April 2018 and all clinical, laboratory and epidemiological data were extracted from this institution's non-Hodgkin's Lymphoma Group Database and electronic medical records. All participants signed an Informed Consent Form, agreeing to participate in this study.

### Study participants

This study included 39 patients with a confirmed diagnosis of SMZL who were followed at our service from January 1992 to December 2016. Patients with mucosal-associated lymphoid tissue extranodal marginal zone lymphoma (MALT) or nodal marginal zone lymphoma (NMZL) with spleen involvement were excluded (overlapping presentations MALT/SMZL and SMZL/NMZL) from analysis.

Clinical and laboratory characteristics assessed at the time of diagnosis and extracted from medical records included: age, gender, staging of Ann Arbor/Cotswolds, B-symptoms, bulky mass, bone marrow infiltration, extranodal involvement sites, performance status by *Eastern Cooperative Oncology Group* (ECOG), transformation to high-grade B-cell NHL, Beta2- microglobulin (B2MG), lactic dehydrogenase (LDH), serum albumin, hemoglobin, leukocytes, lymphocytes, platelet count, HIV, hepatitis B and C serology, presence of monoclonal peak in electrophoresis of serum proteins, leukemization, presence of villous lymphocytes on cytomorphology of peripheral blood smear, presence of paraneoplastic autoimmune phenomena (autoimmune hemolytic anemia, autoimmune thrombocytopenia, reactive arthritis and leukocytoclastic vasculitis), Arcaini score and SMZL Working Group score.

Date of diagnosis, remission, relapse, beginning and end of primary therapy, date of death, cause of death, date of last outpatient evaluation, and type of response achieved after first-line treatment were also computed. Based on this survey we were able to predict the primary outcomes overall survival (OS) and progression-free survival (PFS).

All patients were staged with neck, chest, abdomen and pelvic tomography, as well as unilateral bone marrow biopsy with immunohistochemical (IHC) study. Patients with lymphocytosis had complementary evaluation with cytomorphological analysis of lymphocytes in peripheral blood smear (Leishman staining) and immunophenotyping by flow cytometry.

### Diagnostic criteria

For diagnostic characterization of SMZL cases we used the criteria of the 2016 World Health Organization Classification (WHO/2016) [2]. The gold standard for diagnosis was based on spleen histology, when splenectomy was performed. Splenic involvement by this lymphoma was considered as infiltration of splenic white pulp by small to medium sized atypical lymphoid cells, with predominantly nodular architectural pattern with mature B-lymphoid immunophenotype determined by IHC study [2, 19].

For patients not submitted to splenectomy the diagnosis was based on the association of clinical and laboratory characteristics, including: (1) - characteristic clinical picture, marked by large splenomegaly and minimal lymphadenopathy, usually restricted to the hepatic and splenic hilum, (2) - morphology and peripheral blood immunophenotype, with hematoscopy demonstrating proliferation of small and mature lymphoid cells with thin polar disposition villi, associated with mature and clonal lymphoid B-cell immunophenotype, determined by immunophenotiping using flow cytometry , and (3) - histological analysis of bone marrow, showing intrasinusoidal infiltration pattern by mature B lymphoid cells.

### Treatment, response assessment and follow-up

Asymptomatic patients with SMZL were conducted under clinical observation (“watchful & waiting approach”). Criteria to indicate specific treatment included: presence of constitutional symptoms (fever, weight loss and night sweats), large or symptomatic splenomegaly, cytopenias such as Hb < 100 g/L, neutrophils < 1.0 x 10^9^/L and platelets < 100 x 10^9^/L and autoimmune manifestations (ITP or AIHA) not responsive to corticosteroids.

Symptomatic SMZL patients received specific treatment and were grouped as follows: (1) - undergoing splenectomy, (2) - undergoing exclusive immunotherapy, comprising administration of the anti-CD20 monoclonal antibody rituximab on a schedule of 375 mg/m^2^ IV once a week, for 4 consecutive weeks (D1, D8, D15 and D22) and (3) - undergoing conventional chemotherapy, including the schemes: chlorambucil 10 mg/m^2^ PO D1 to D6 30/30 days, CVP (cyclophosphamide 750 mg/m^2^ IV D1 , vincristine 1.4 mg/m^2^ [Max 2.0 mg] IV D1 and prednisone 100 mg PO D1-D5 21/21 days) or fludarabine monotherapy 40 mg/m^2^ PO D1-D5 30/30 days.

Response to the employed therapy was accessed based on the 2014 *Lugano Response Criteria* [20], based on clinical laboratory, tomographic and histopathological criteria (bone marrow reevaluation in patients with bone marrow infiltration at the time of diagnosis). At the end of treatment, patients were monitored with clinical and laboratory examination every 3 months in the first year, every 4 months in the second year, and every 6 months after the third year.

### Statistical analysis

The univariate analysis to assess the association among categorical variables was performed using Mantel-Haenszel chi-square test. A Cox univariate analysis was performed to estimate the association between categorical variables and survival curves, and thus determine the factors with prognostic implication. The log-rank test was used to compare survival curves and to verify the association between categorical variables and survival curves.

Overall survival (OS) and progression-free survival (PFS) curves were estimated by the Kaplan-Meier method. OS was measured from the date of diagnosis to the date of death from any cause and was censored at the date of the last follow-up. PFS was assessed from the date of the diagnosis to the date of progression, death from any cause, or the last follow-up.

The determination of cutoff points for hemoglobin and platelet counts were based on current literature (Hb 100 g/L and platelets 100 x 10^9^/L). LDH, B2-microglobulin and albumin values were based on the higher normality value of the commercial kits for these tests used in the laboratory routine of our hospital (480 U/L for LDH, 1.7 mg/dL for B2MG and 3.5 g/dL for albumin). Cutoff points for leukocyte and lymphocyte counts were based on the determination of the median for respective variables (6.1 x 10^9^/L for leukocytes and 2.7 x 10^9^/L for lymphocytes). Statistical analysis was performed using the software STATA 12.0 and a value of p ≤ 0.05 was considered statistically significant.

## Results

### Clinical-laboratory and epidemiological characteristics

A total of 42 patients with splenic lymphoma were initially identified. After reviewing medical records 3/42 (7.1%) were excluded because they presented overlapping characteristics with nodal marginal zone lymphoma (SMZL/NMZL). Table 1 lists the characteristics of the 39 SMZL patients included in the analysis. The median age was 63 years (range 28 - 76 years) and 71.8% (28/39) were female.

**Table 1:** Clinical-laboratory and epidemiological characteristics of 39 SMZL patients

Characteristics	Value
Age (years)	63 (28-76)
Gender	
Female	28 (71.8%)
Male	11 (28.2%)
Ann Arbor stage	
I	05 (12.8%)
IV	34 (87.1%)
ECOG	
0 a 1	23 (58.9%)
2 a 4	16 (41.0%)
Extra-splenic involvement	34 (87.1%)
B symptoms	22 (56.4%)
Bone marrow involvement	30 (76.9%)
Peripheral blood involvement	17 (43.5%)
Transformation to high grade NHL	04 (10.2%)
Auto-immune manifestations	11 (28.2%)
Hepatitis B virus positive	04 (10.2%)
Hepatitis C virus positive	02 (5.1%)
Monoclonal paraprotein	07 (17.9%)
Primary treatment	
Rituximab monotherapy	08 (20.5%)
Splenectomy	21 (53.8%)
Chemotherapy	09 (23.0%)
Watchful & waiting	01 (2.5%)
Hemoglobin (g/L)	109 (range 62-160)
Leukocytes (x 10^9^/L)	6.1 (range 0.5-24.4)
Lymphocytes (x 10^9^/L)	2.7 (range 0.4-22.3)
Platelets (x 10^9^/L)	118 (range 4-455)
LDH (U/L)	438.5 (range 135-1276)
Albumin (g/dL)	4.1 (range 1.7-4.9)
B2 microglobulin (mg/dL)	3.8 (range 1.5-25.0)

Most patients had advanced stage (Ann Arbor/Cotswolds IV) and 58.9% (23/39) had good performance status (ECOG 0 or 1). Extra-splenic involvement was observed in 87.2% (34/39), especially bone marrow involvement in 76.9% (30/39) and peripheral blood in 43.5% (17/39). Among patients with circulating phase, 47% of these (8/17) had small villous lymphocytes in the morphology of the peripheral blood smear. B-symptoms were observed in 56.4% of cases (22/39).

High-grade B-cell NHL transformation occurred in 10.2% (4/39). Serology for C hepatitis was positive in 5.1% (2/39) and for B hepatitis in 10.2% (4/39). Monoclonal protein was observed in 17.9% (7/39) and paraneoplastic phenomena of immune nature occurred in 28.2% of the cases (11/39), especially autoimmune hemolytic anemia and autoimmune thrombocytopenia.

Regarding the laboratory parameters, the medians values were: Hemoglobin (g/L) 109 (range: 62 - 160); leukocytes (x 10^9^/L) 6.1 (range: 0.4 - 24.4); lymphocytes (x 10^9^/L) 2.7 (range: 0.4 - 22.3); platelets (x 10^9^/L) 118 (range: 4-455); LDH (U/L) 438.5 (range: 135-1276); B2MG (mg/dL) 3.8 (range: 1.5 - 25.0) and albumin (g/dL) 4.1 (range: 1.7 - 4.9) (Table 1).

The decision of exclusive observation (W&W approach) was taken in only 2.5% (1/39) of SMZL patients. As part of the initial treatment 20.5% (8/39) received immunotherapy alone, with antiCD20 monoclonal antibody rituximab weekly, for 4 consecutive weeks. Splenectomy was the primary treatment in 53.8% (21/39), while 23.0% (9/39) were initially treated with conventional single-drug or combination chemotherapy (chlorambucil, CVP or fludarabine regimens). The Table 2 summarizes the main clinical, laboratory and epidemiological baseline characteristics stratified by therapeutic intervention group: Group A – splenectomy (*N*=21), Group B – rituximab monotherapy (*N*=08) and Group C – low-intensity mono or polychemotherapy (*N*=09).

**Table 2:** Comparison of clinical and laboratory characteristics of SMZL patients stratified by type of primary treatment

Variable	Grup A Splenectomy (*N*=21)	Grup B Rituximab (*N*=8)	Grup C Chemotherapy (*N*=9)	*p*-value
Age (median, range)	61 (41-76)	54 (33-71)	65 (28-76)	0.194
Β2-microglobulin (median)	2.95 mg/dl	4.15 mg/dl	4.75 mg/dl	**0.048**
LDH (median)	388 U/L	452 U/L	535 U/L	**0.050**
Albumin (median)	4.20 g/dl	3.65 g/dl	3.80 g/dl	**0.005**
Hemoglobin (median)	110 g/L	91 g/L	100 g/L	0.340
Platelets (median)	118 x 10^9^/L	115 x 10^9^/L	112 x 10^9^/L	0.213
Comorbities ≥ 2	14.3 %	100 %	44.4 %	**0.00024**
ECOG ≥ 2	9.5 %	75 %	55.5 %	**0.050**
Arcaine int/high-risk	33.3 %	62.5 %	44.4 %	0.116
SMZL-WG int/high-risk	76.2 %	100 %	100 %	**0.030**

B2-microglobulin: A x B *p*=0.047, A x C *p*=0.038, B x C *p*=0.274

LDH: A x B *p*=0.050, A x C *p*=0.023, B x C *p*=0.170

Albumin: A x B *p*= 0.030, A x C *p*=0.048, B x C *p*=0.176

Comorbities: A x B *p*= 0.0001, A x C *p*=0.025, B x C *p*=0.00034

ECOG: A x B *p*= 0.002, A x C *p*= 0.037, B x C *p*=0.164

SMZL/WG: A x B *p*=0.023, A x C *p*=0.023, B x C *p*=0.547

Second-line treatment for relapsed/refractory (R/R) disease was used in 28.2% (11/39) of cases. Among these 11 cases all had recurrent disease, that is, none of them behaved as a primary refractory disease to the initially employed therapeutic modality. The median time between the end of the first therapeutic line and initiation of second line treatment was 23 months (range 7.3 - 187 months). Two cases (2/11 - 18.2%) which received monotherapy with rituximab in the first line had late disease progression (> 12 months) and underwent splenectomy. They reached a partial response and after a short period of time they died due to disease progression.

Six patients (6/11-54.6%) initially splenectomized progressed the disease symptomatically, being treated with rituximab monotherapy as rescue, five of whom (5/6-83.4%) presented sustained CR and one (1/6-16.6%) PR with rapid disease progression, culminating in death attributable to lymphoma. Three patients (3/11 - 27.3%) who initially received first-line therapy with low-intensity cytotoxic chemotherapy (CVP protocol – cyclophosphamide, vincristine and prednisone), progressed rapidly (< 12 months) after this treatment, but were adequately rescued with rituximab monotherapy, reaching sustained CR until this moment.

### Survival outcomes

**Fig. 1:**
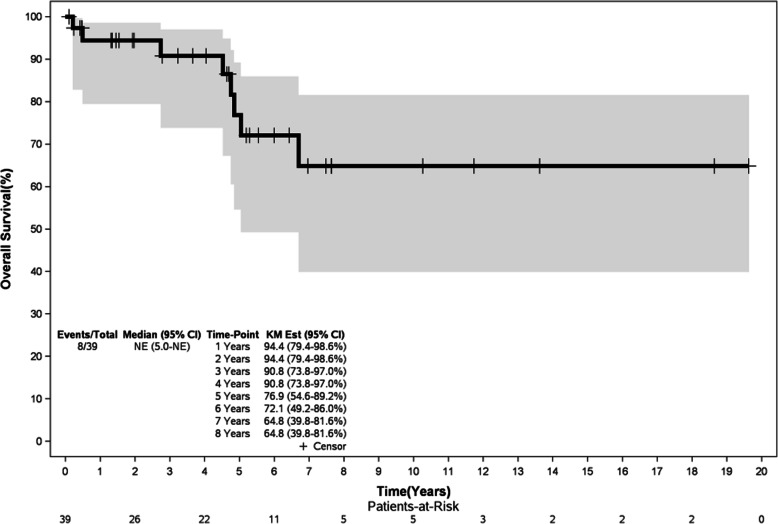
Overall survival curve for 39 SMZL Brazilian patients

**Fig. 2:**
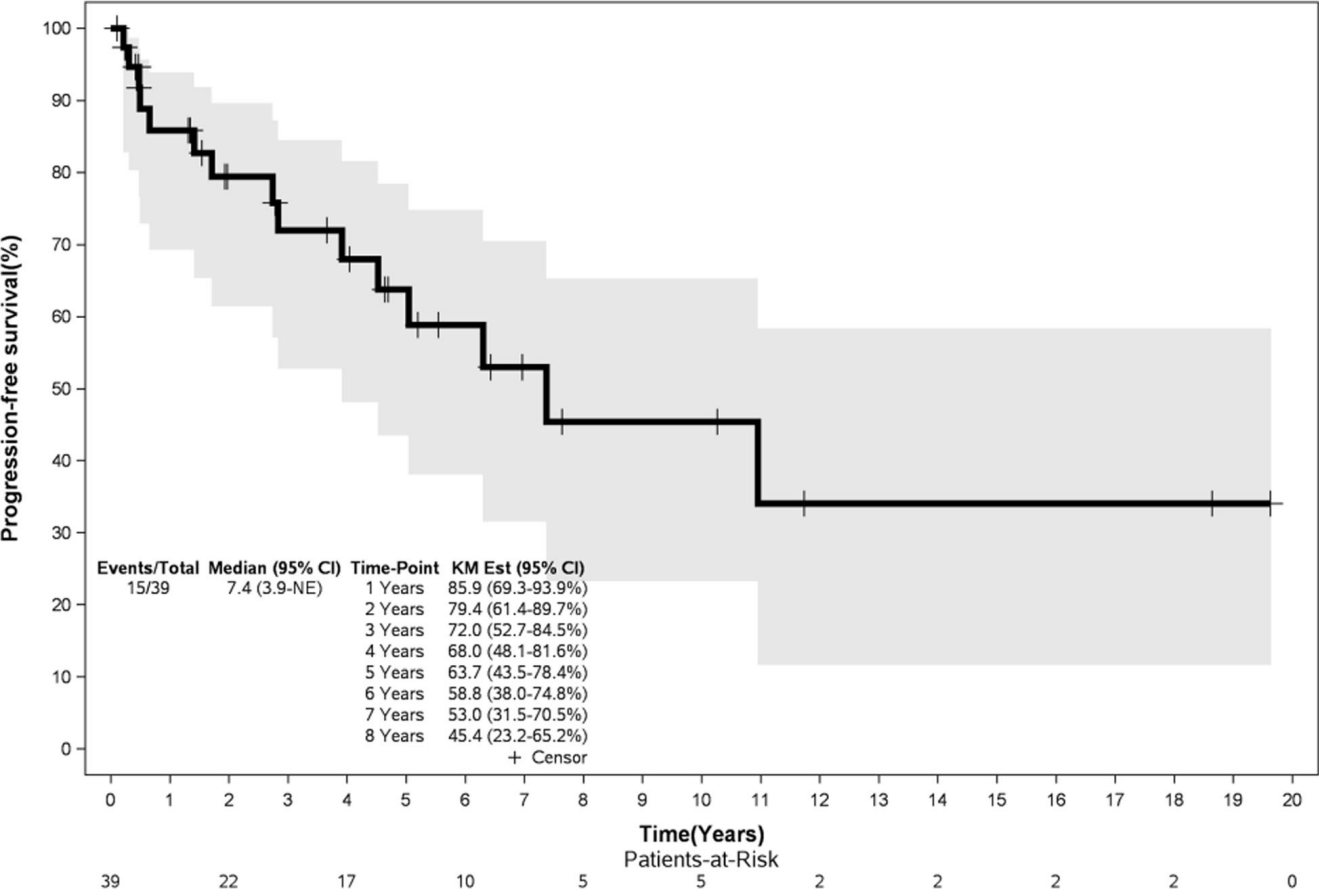
Progression-free survival for 39 SMZL Brazilian patients

At median follow-up of 8.7 years (0.6 – 20.2 years), the median OS was not reached yet and it was 7.4 years for PFS. The estimated 5-year OS was 76.9% (Figure 1) and 63.7% for PFS (Figure 2). There was no statistically significant difference for OS and PFS between the three treatment groups, as summarized in Table 3.

**Table 3:** Median OS and PFS for 39 SMZL patients separated by treatment modality

Treatment	Median OS (CI 95%)	Median PFS (CI 95%)
Rituximab	5.9 year (3.8-7.9)	6.0 year (4.3-7.7)
Splenectomy	13.9 year (8.4-19.4)	10.9 year (5.7-16.2)
Chemotherapy	13.5 year (5.8-21.2)	15.1 year (10.3-19.9)
***p*-value**	**0.565**	**0.530**

Eight patients died at last follow up, and at 10-years the cumulative incidence of mortality was 20.5% (8/39); disease progression was the main cause of death in 6 out of 8 patients (75%) (6/8), one patient died of infection complications (12.5%) (1/8) and another of cardiovascular complication (12.5%) (1/8).

### Determination of prognostic factors

To estimate prognosis we applied the Arcaini and *SMZL Working Group* (SMZL WG) scores on the population studied. By the Arcaini score, including Hb < 120 g/L, LDH > NSV and albumin < 3.5 g/dL variables, 17.9% (7/39) patients were classified in low risk (0 factor), 41.0% (16/39) as intermediate risk (1 factor) and 41.0% (16/39) in high risk (≥ 2 factors). When SMZL-WG was used, 12.8% (5/39) were stratified into low-risk (0 factors), 66.6% (26/39) in intermediate risk (1 or 2 factors) and 20.5% (8/39) in high-risk (3 or 4 factors). This score included the variables Hb < 95 g/L, platelets < 80 x 10^9^/L, LDH > NSV and lymph node involvement outside of the splenic hilly.

**Fig. 3:**
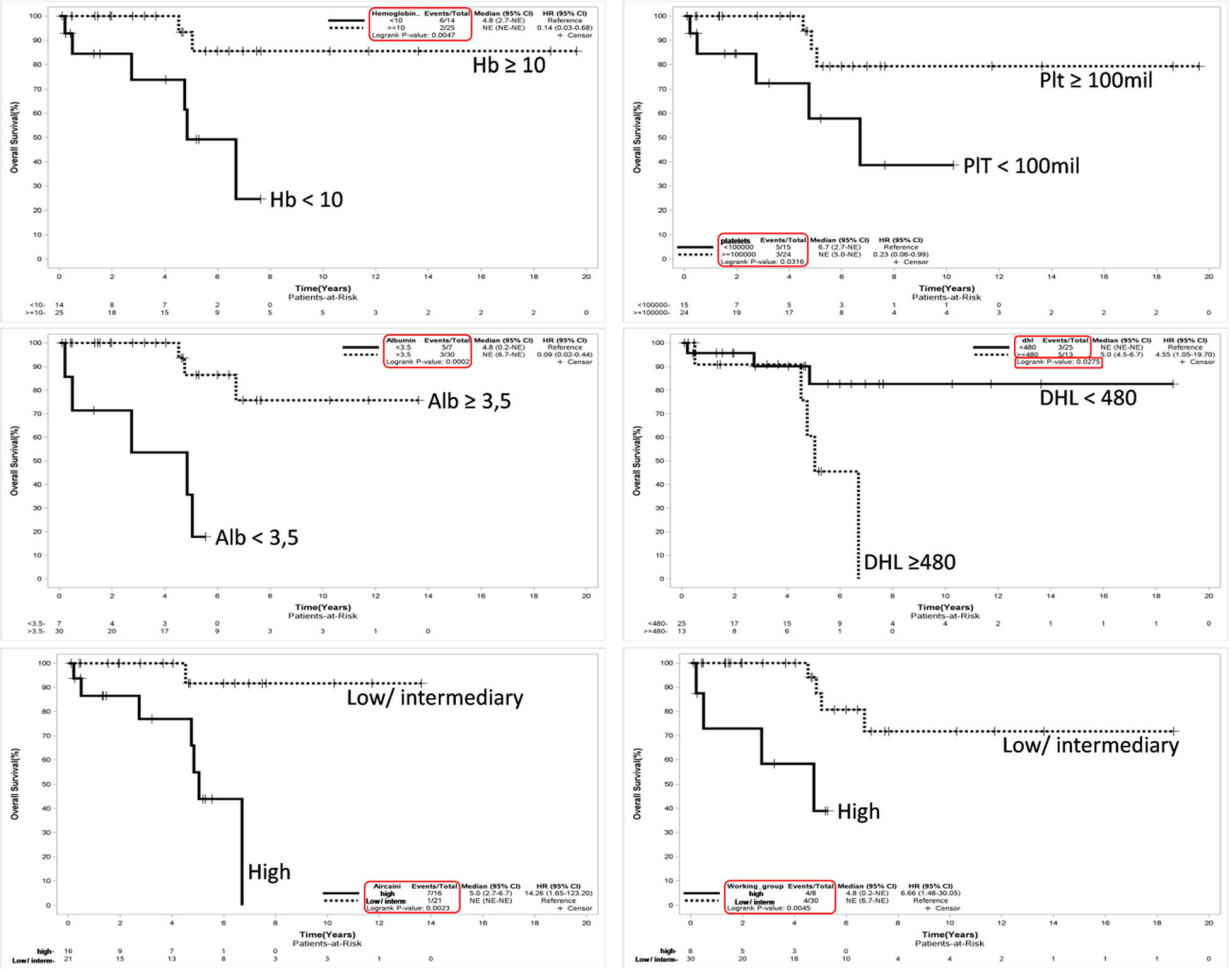
OS curves according hemoglobin, platelets count, albumin, LDH, Arcaine risk score and *SMZL Working Group risk* score

In univariate analysis, the prognostic factors associated with worse 5-year OS were: LDH ≥ 480 U/L (HR: 4.55, 95% CI 1.05-19.70, *p*=0.043), serum albumin < 3.5 g/dL (HR: 7.32, 95% CI 1.46-36.32, *p*=0.015), platelets < 100 x 10^9^/L (HR: 7.32, 95% CI 2.26-60.54, *p*=0.003), hemoglobin < 100 g/L (HR: 4.27, 95% CI 1.01-17.99, *p*=0.048), high-risk Arcaini score (HR: 14.26, 95% CI 1.65-123.20, *p*=0.002) and SMZL-WG high-risk score (HR: 6.66, 95% CI 1.48-30.05, *p*=0.004). Furthermore, the clinical stage IV (*p*=0.731), bone marrow involvement (*p*=0.548), elevated Beta2- microglobulin (0.375), leukocytes ≥ 6.1 x 10^9^/L (*p*=0.432), lymphocytes ≥ 2.7 x 10^9^/L (*p*=0.237) and ECOG ≥ 2 (*p*=0.136) were not related to poor OS. Figure 3 shows OS curves according to variables that were statistically significant.

**Fig. 4:**
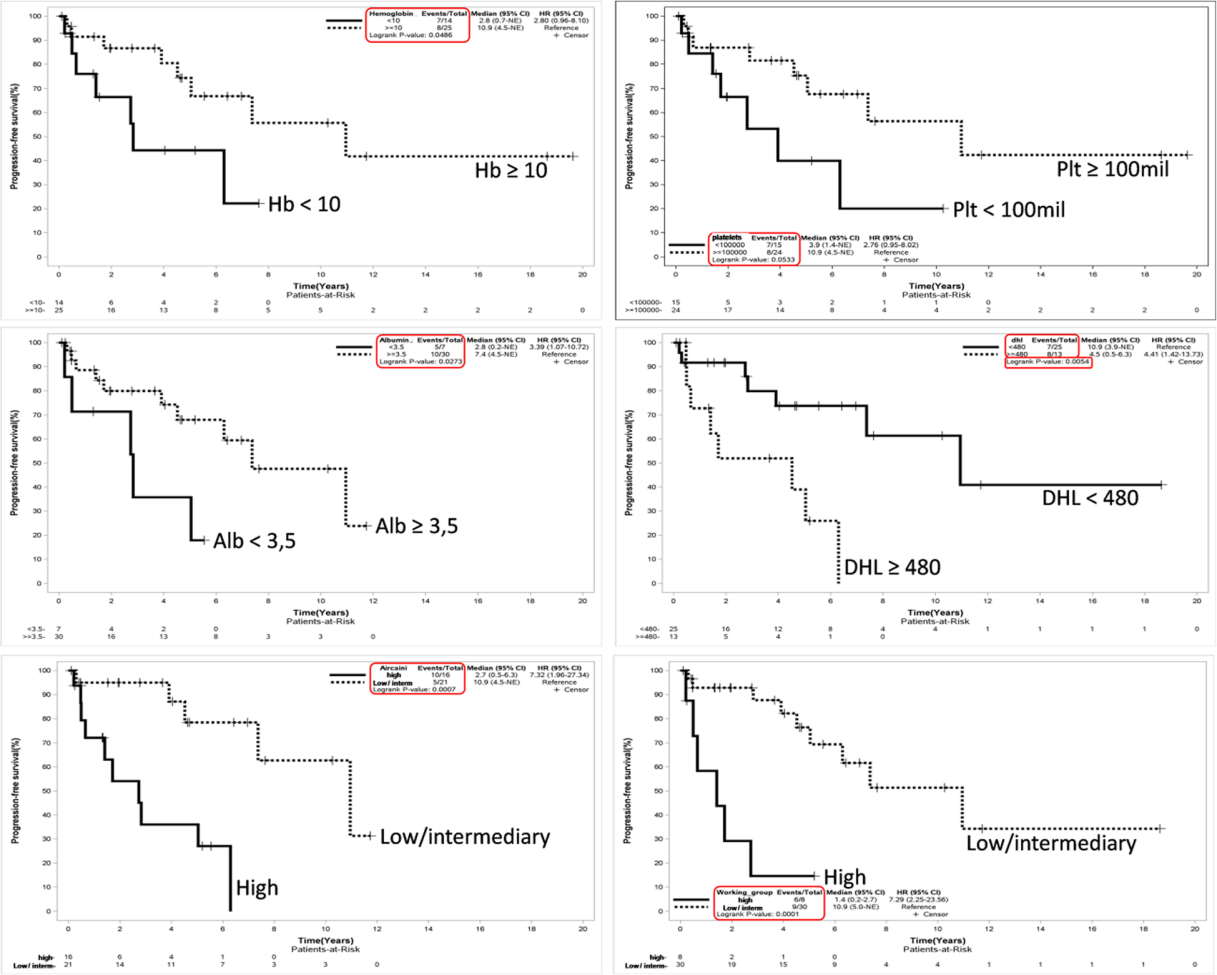
PFS curves according hemoglobin, platelets count, albumin, LDH, Arcaine risk score and *SMZL Working Group* risk score

Similarly, LDH ≥ 480 U/L (HR: 4.47, 95% CI 1.42-13.73, *p*=0.005), albumin < 3.5 g/dL (HR: 3.39, 95% CI 1.07-10.72, *p*=0.007), hemoglobin < 100 g/L (HR: 2.80, 95% CI 0.95-8.10, *p*=0.048), platelets < 100 x 10^9^/L (2.78, 95% CI 0.95-8.02, *p*=0.050), high risk Arcaini score (HR: 7.32, 95% CI 1.96-27.34, *p*=0.0007), and high-risk SMZL-WG score (HR: 7.29, 95% CI 2.25-23.56, *p*=0.001) were associated with worse 5-year PFS. The clinical stage IV (*p*=0.386), bone marrow involvement (*p*=0.945), elevated Beta2-microglobulin (*p*=0.321), leukocytes ≥ 6.1 x 10^9^/L (*p*=0.657), lymphocytes ≥ 2.7 x 10^9^/L (*p*=0.549) and ECOG ≥ 2 (*p*=0.945) were also not associated with poor PFS. Figure 4 shows PFS curves according to variables that were statistically significant.

## Discussion

Splenic marginal zone lymphoma (SMZL) is a rare B-cell lymphoid malignancy, representing 2% of all non-Hodgkin's lymphomas and 80% of all primary spleen neoplasms [2]. The natural history and clinical-biological characteristics of this disorder are known from case series reports from some European and North American centers [15, 16, 18]. Here, we had the opportunity to describe the characteristics of this tumor in a population derived from a high degree of miscegenation, from a different geographical area, and that may have distinct biological findings. To our knowledge, this is the larger cohort of Latin American SMZL patients reported in the medical literature.

In our cohort, we found a predominance of SMZL in females, with a 3:1 distribution ratio [15, 18], contrarily to what has been observed in series of patients reported from developed countries. Although we believe that women are more careful about their own health and seek medical help earlier than men, particularly in unfavorable socio-economic conditions, we cannot ignore these findings. In agreement with our study, Xing et al. reported 107 cases of SMZL treated at the *British Columbia Cancer Agency* (BCCA), also observing a predominance of this cancer in women, with a distribution ratio of 1.5: 1.0 [16].

B-symptoms and higher rates of involvement of extralymphonodal sites, particularly bone marrow and peripheral blood, were more frequent in our cohort. We believe that these findings may be explained by the increased difficulty in accessing tertiary health services and referral cancer centers in Brazil, as well as in other developing countries. This may justify the larger tumor mass and incidence of B symptoms attributed to disease diagnosed in later stages.

In our series, we found a rate of 10.2% (04/39) for high-grade B-cell NHL histological transformation, similar to that observed in the BCCA (15/107 - 14%) and *Sylvester Comprehensive Cancer Center of Miami* cohorts (34/453 - 7.5%) [16, 17]. In the Brazilian cohort all cases of transformation were documented by biopsy. Due to our limited sample (N = 39) we could not access predictors of transformation, but we noted the unfavorable outcome associated with this phenomenon, as all patients with transformed disease died in our cohort. Alderuccio et al., analyzing 453 patients with marginal zone lymphoma (MALT, nodal and splenic), identified failure to achieve CR after initial treatment, elevation of lactic dehydrogenase and more than 4 nodal sites involved at the time of diagnosis as predictors of increased risk of MZL transformation [17]. Overall, prognosis after transformation is quite narrow, with 5-year OS of 0% in our Brazilian patients, 33% in BCCA and 43% in the North-American group [16, 17].

The etiological association between SMZL and chronic infection for hepatitis C virus is commonly related in Southern Europe, and the development of lymphoma under these conditions appears to be associated with activation of CD81 in B-cells by chronic stimulation of the virus E2 glycoprotein [21, 22]. According to some reports, up to 30% of SMZL patients may have chronic hepatitis C [7, 21, 22]. These cases may initially be treated with HCV-guided therapy, such as interferon alpha and ribavirin or a combination of new antiviral agents, and many cases may have remission of lymphoma with such therapies. In our series, 5.1% (2/39) of SMZL patients had positive serology for hepatitis C, but surprisingly 10.2% (4/39) had chronic hepatitis B infection. Recently, some studies have shown coexistence between chronic hepatitis B virus infection and splenic lymphomas, particularly SMZL, suggesting a new etiological association for this neoplasm. Similar to that observed in HCV positive cases, treatment for HBV with antiviral agents is capable of inducing neoplasm remission [23–26].

SMZL is usually an indolent and chronic disease, with a median overall survival exceeding 10 years [2, 15, 16, 18]. In this study, we showed quite prolonged OS and PFS (5-year OS 76.9% and 5-year PFS 63.7%), estimated over several years, similar to what is reported in the literature. However, 20%-30% of SMZL patients are characterized by greater biological aggressiveness, with shortened OS (median 4 years) and greater propensity for histopathological transformation to high grade B-cell lymphoma [15]. Therefore, identifying this subgroup of ominous outcome is essential to choose the best treatment options, particularly in resource-poor settings where the best therapy is not always universally accessible.

To this end, different international collaborative groups analyzed their own series of SMZL patients, seeking to identify factors associated with unfavorable prognosis, as well as to elaborate prognostic scores for outcome prediction and implementation of risk-adapted therapies. In 2006, Arcaini et al. analyzed 309 SMZL patients and identified 3 laboratory variables that directly impacted OS and PFS, with Hb < 120 g/L, LDH > UNV and albumin < 3.5 g/dl, and through a simple scoring system created a mortality predictor prognostic score [18].

Following the same principles, in 2012 a multicenter study involving 593 SMZL patients from different international cancer centers, known as the *Splenic Marginal Zone Lymphoma Working Group* (SMZL-WG), determined the unfavorable predictor value of Hb < 95 g/L, platelets < 80 x 10^9^/L, LDH > UNV and presence of extra hilum-splenic lymphadenopathy for symptomatic progression-free survival at 5 years. The SMZL-WG score was thus created, stratifying patients at low-risk, intermediate-risk and high-risk, with 5-year PFS of 95%, 87% and 68%, respectively [15].

Although this is the largest Latin-America single-center cohort of SMZL patients published to date, our study had only 39 cases, which imposes some restrictions on the interpretation of our findings. However, we were able to statistically determine variables associated with worse OS and PFS at 5 years, and we could validate the use of Arcaini and SMZL-Working Group scores in our population. Prognostic factors in the Brazilian series involved Hb < 100 g/L, platelets < 100 x 10^9^/L, LDH > UNV and albumin < 3.5 g/dL. These laboratory prognostic factors were similar to those described in the medical literature, but we could not create a prognostic score using such variables due to the limited N of our study.

The therapeutic approach of SMZL has shown substantial progress in the last 15 years. As with other indolent lymphomas, asymptomatic patients do not benefit from early treatment and should only be observed with a “watchful & waiting” approach [9, 19]. However, treatment should be introduced in the presence of B symptoms, massive or symptomatic splenomegaly, cytopenias secondary to hypersplenism or bone marrow lymphomatous infiltration, or unresponsive-corticosteroid immune manifestations [9, 19].

Until 2005, splenectomy was considered the first-line therapy for SMZL (“gold-standard”). Although it is an effective therapy, with an overall response rate (ORR) of almost 90% and PFS of more than 50% in 5 years, the morbidity and mortality associated with the surgical procedure are not negligible, affecting about 5% of patients [27–29]. After splenectomy, splenomegaly-related symptoms resolve and cytopenias improve. Furthermore, histological analysis of the spleen will allow the diagnosis of SMZL to be confirmed. However, it should be emphasized that this therapeutic strategy is not curative and that most patients will achieve only partial response, with persistent bone marrow and peripheral blood lymphomatous infiltration, even if a significant reduction in lymphocytosis was observed [28].

Among chemotherapy options, different studies have shown that alkylating agents alone, such as chlorambucil, cyclophosphamide or in combination (CVP, CHOP) are associated with low response rates and high toxicity, considering the indolent behavior of the disease. On the other hand, purine analogs, particularly fludarabine, are associated with higher overall response rates (100%) and complete response (70%) [30–32].

After 2005, incorporation of the anti-CD20 monoclonal antibody (rituximab) changed the therapeutic paradigm of SMZL patients. The weekly regimen of rituximab monotherapy at a dose of 375 mg/m^2^ for 4 to 6 consecutive weeks is associated with minimal toxicity and OS and PFS ranging from 89%-100% and 73%-98%, respectively [12, 13, 33]. In addition, rituximab is also effective for autoimmune disorders such as AIHA and immune thrombocytopenia, found in up to 15-20% cases of SMZL.

Although large prospective and randomized studies comparing rituximab monotherapy versus splenectomy are still lacking, the Greek group, retrospectively, compared rituximab monotherapy versus splenectomy in a historical cohort and found 5-year OS and PFS of 94% vs 77% (p = 0.09) and 72% vs 58% (p = 0.09) for rituximab [13]. In 2018, the same group published an update of their data (n = 108), confirming the high efficacy of rituximab monotherapy, its role as a splenectomy-sparing therapeutic strategy and the potential benefit of maintenance treatment with immunotherapy. In this study, responses to rituximab were long lasting, with 10-year PFS exceeding 60%, with minimal toxicity, and maintenance therapy improved response quality and was associated with better PFS [14].

Another strategy associated with a high success rate in the treatment of SMZL is a chemo-immunotherapy approach with bendamustine/rituximab (BR) regimen. According to recent studies, this association provides high overall and complete response rates (91% and 73%, respectively), with manageable toxicity and unquestionable benefit, particularly in SMZL patients with intermediate and high-risk scores [34, 35]. Faced with the unquestionable benefits of rituximab monodrug or BR combination chemoimmunotherapy, some authors propose that splenectomy may be abandoned in the up-front management of SMZL [36]. We believe that splenectomy still plays an important therapeutic role in the management of SMZL, particularly in patients not responsive to immunotherapy or chemotherapy and as up-front modality in those with confined spleen disease, in which this is a diagnostic and therapeutic procedure that occurs in about 15%-20% of SMZL patients.

Our cohort comprised SMZL patients treated at our service for about 25 years, therefore, the therapeutic strategies used were quite heterogeneous, including chemotherapy (single or combined), splenectomy and rituximab immunotherapy. In our series, none of these therapeutic approaches showed statistically significant superiority in terms of OS or PFS, but it should be noted that in a context of poor-resource settings, such as in the Brazilian public health system, rituximab has been reserved for cases with known worse prognosis, such as elderly patients or those with high surgical risk and adverse prognostic factors. In addition, our study has restrictions regarding the small casuistic and short segment time of patients receiving immunotherapy (incorporated in our institution for reserved cases of SMZL starting in 2008).

As demonstrated by the data in Table 2, some baseline characteristics differ in statistical significance form (p < 0.05) between the three primary treatment groups. In general, these differences are strongly expressed between splenectomized (*N*=21) and non-splenectomized (*N*=17, rituximab or low-intensity cytotoxic chemotherapy) patients.

Patients undergoing splenectomy had lower levels of tumor markers (B2-microglobulin and LDH), thus inferring a smaller tumor mass than patients treated with rituximab or cytotoxic chemotherapy. In addition, surgically treated patients had better general clinical conditions than the non-splenectomized group, reflected by higher levels of serum albumin, lower rate of clinical comorbidities and better performance status according to the Eastern Cooperative Oncology Group (ECOG) scale. Finally, the group undergoing splenectomy had a lower proportion of patients classified as intermediate or high-risk by the prognostic index of the SMZL-Working Group compared to patients treated with medications.

Thus, we noticed that SMZL patients who received immunotherapy had worse general clinical conditions, and laboratory characteristics were consistently associated with a poor prognosis, which led us to infer that this is one of the reasons why we did not find a concrete benefit in terms of OS and PFS of the rituximab group versus splenectomy group in our cohort.

## Conclusion

In conclusion, based on the risk factors identified in our real-life study and according to the latest evidence available in the literature for SMZL therapy, we propose that in resource-poor settings, where access to rituximab therapy is not universal, as is the case in the Brazilian public health system, a risk-adapted therapeutic approach should be employed in the management of SMZL patients (Table 4). Thus, rituximab may be reserved for first-line therapy in elderly patients, in those with high operative risk or who have one or more of the following factors: Hb < 100 g/L, < 100 x 10^9^/L platelets, LDH > UNV, albumin < 3.5 g/dL or high-risk Arcaini and SMZL-WG scores. Patients who do not present these factors can be satisfactorily approached with splenectomy, when therapy is indicated.

**Table 4:** Risk-adapted approach for treatment of SMZL patients in resource-poor settings

Clinical condition	Suggested approach
1 – Asymptomatic SMZL	“Watchful & waiting”
2 – Symptomatic SMZL	
2.1. With hepatitis C co-infection	2.1. Interpheron alpha and ribavirin, or new antiviral drugs
2.2. Age < 65 years, no-comorbidities and low-risk (Hb > 100 g/L, > 100 x 10^9^/L platelets, LDH < 480 U/L and albumin > 3.5 g/dL)^a^	2.2. Splenectomy
2.3. Age > 65 years, comorbidities or high-risk (Hb < 100 g/L, < 100 x 10^9^/L platelets, LDH > 480 U/L and albumin < 3.5 g/dL)^a^	2.3. Rituximab weekly for 4 weeks
3 – Relapsed/Refractory SMZL	
3.1. Not exposed to rituximab	3.1. Rituximab weekly for 4 weeks
3.2. Exposed to rituximab	3.2. Splenectomy (if not done) or fludarabine (4 to 6 cycles)
4 – High-grade B-cell NHL transformation	4. 6 to 8 cycles of R-CHOP +/- autologous SCT^b^

^a^presence of any adverse factors

^b^consider auto-SCT particularly after previous exposition to rituximab

## Data Availability

All data generated and analysed during this study are included in this published article. The raw data that generated the results presented in this paper are part of the Database of the Group of Non-Hodgkin's Lymphomas at the University of São Paulo and can be made available upon request to the correspondence author via e-mail.

## Abbreviations

SMZL: Splenic marginal zone lymphoma
NHL: Non-Hodgkin lymphoma
OS: Overall survival
PFS: Progression-free survival
SMZL-WG: Splenic marginal zone lymphoma working group
Hb: Hemoglobin
LDH: Lactate dehydrogenase
MALT: Mucosa associated lymphoid tissue
NMZL: Nodal marginal zone lymphoma
ECOG: Eastern Cooperative Oncology Group
B2mg: Beta2- microglobulin
WHO: World Health Organization
ITP: Immune thrombocytopenic purpura
AIHA: Autoimmune hemolytic anemia
CVP: Cyclophosphamide, vincristine and prednisone
W&W: Watchful & waiting
R/R: Relapsed/refractory disease
NSV: Normal superior value
HR: Hazard ratio
CI: Confidence interval
BCCA: British Columbia Cancer Agency
CR: Complete response
HCV: Hepatitis C virus
HBV: Hepatitis B virus
UNV: Upper normal value
CHOP: Cyclophosphamide, doxorubicin, vincristine and prednisone
BR: Bendamustine and rituximab
